# Monitoring effectiveness of nirsevimab immunization against RSV hospitalization using surveillance data: a test-negative case–control study, Spain, October 2024–March 2025

**DOI:** 10.1017/S0950268825100782

**Published:** 2025-12-09

**Authors:** Sandra Campos Mena, Gloria Pérez-Gimeno, Nicola Lorusso, Virginia Álvarez Río, Luca Basile, Noa Batalla Rebollo, Luis García-Comas, Blanca Andreu Ivorra, Jordi Pérez-Panadés, Violeta Ramos Marín, Daniel Castrillejo, Ana Fernández Ibáñez, María Ángeles Rafael de la Cruz López, Olivier Núñez, Susana Monge, the SiVIRA group

**Affiliations:** 1 National School of Health, Institute of Health Carlos III, Madrid, Spain; 2 National Distance Education University (UNED), Madrid, Spain; 3 CB06/02/0085, Biomedical Research Networking Centre on Epidemiology and Public Health (CIBERESP), Madrid, Spain; 4 Department of Communicable Diseases, Centre of Epidemiology, Carlos III Health Institute (CNE-ISCIII), Madrid, Spain; 5 Health and Consumption Department, General Directorate of Public Health and Pharmaceutical Management of Andalusia, Spain; 6 Epidemiology Service, General Directorate of Public Health, Castilla y León, Spain; 7 Department of Health, Public Health Agency of Catalonia (ASPCAT), Catalunya, Spain; 8 Subdirectorate of Epidemiology, General Directorate of Public Health, Extremadura, Spain; 9 Communicable Diseases Area, General Subdirectorate of Public Health, General Directorate of Public Health, Madrid, Spain; 10 Department of Epidemiology, Regional Health Council, IMIB, Murcia, Spain; 11 General Subdirectorate of Epidemiology, Health Surveillance and Environmental Health, General Directorate of Public Health, Comunitat Valenciana, Spain; 12 Epidemiological Surveillance Service, General Directorate of Public Health, Ceuta, Spain; 13 Epidemiological Surveillance Service, General Directorate of Public Health, Melilla, Spain; 14 Epidemiological Surveillance Section, General Directorate of Public Health and Mental Health, Asturias, Spain; 15 Epidemiology Service, General Directorate of Public Health, Castilla-La Mancha, Spain; 16 CB21/13/00091, Biomedical Research Networking Centre on Infectious Diseases (CIBERINFEC), Madrid, Spain

**Keywords:** respiratory syncytial virus, infant, immunization, nirsevimab, surveillance system, test-negative design

## Abstract

Effectiveness of nirsevimab against respiratory syncytial virus (RSV) hospitalization during the 2024/2025 season in Spain was estimated using a test-negative design (TND) and hospital-based respiratory infections surveillance data. Children born between 1 April 2024 and 31 March 2025 and hospitalized with severe respiratory infection between the start of the 2024 immunization campaign (regionally variable, between 16 September and 1 October 2024) and 31 March 2025 were systematically RT-PCR RSV-tested within 10 days of symptom onset and classified as cases if positive or controls if negative. Nirsevimab effectiveness ((1 − odds ratio) × 100) was estimated using logistic regression, adjusted for admission week, age, sex, high-risk factors, and regional RSV hospitalization rate. We included 199 cases (68.8% immunized) and 360 controls (86.4% immunized). Overall effectiveness was 65.5% (95% confidence interval: 45.2 to 78.3). Effectiveness was similar among infants born before and after the campaign start (63.6% vs. 70.4%, respectively). We found an unexpected early decrease in effectiveness with increasing time since immunization and age, albeit with wide confidence intervals for some groups. Strong age–period–cohort effects and potential sources of bias were identified, highlighting the need to further explore methodological challenges of implementing the TND in the dynamic population of newborns.

## Key results

- Nirsevimab immunization of infants born or entering their first RSV season was first implemented in the 2023–2024 season in Spain, and its effectiveness needs to be monitored over time.
- Overall effectiveness of nirsevimab against RSV hospitalization, estimated using a test-negative case–control design in the 2024/2025 season, was 65.5% (95% CI: 45.2 to 78.3), slightly lower than estimates from the first season of nirsevimab implementation, mostly estimated with population based studies.
- Effectiveness against RSV hospitalization was mostly similar in infants born before the campaign start (catch-up group) and those born afterward (at-birth immunization group), at 63.6% versus 70.4%, respectively.
- Unexpected early decrease in effectiveness by time since immunization may show the influence of true decrease in protection, random variation, given the low statistical power for some stratified analyses, and/or potential biases.

## Introduction

Respiratory syncytial virus (RSV) is one of the leading causes of hospitalization in infants, with risk declining with increasing age [1, 2]. Nirsevimab, a monoclonal antibody approved for the prevention of RSV severe infection in infants entering their first respiratory season, was first recommended in Spain in October 2023 [3].

In its first season of implementation, nirsevimab achieved high uptake, with coverage around 90% [4]. Observational data from this period indicated substantial effectiveness and impact, with estimated reductions in RSV-related hospitalizations ranging from 70% to 90% [5–7], comparable to the efficacy reported in randomized controlled trials (77% and 83%) [8–10].

However, many of those studies relied on resource-intensive study designs, such as population based or matched case–control and cohort studies, which may limit their scalability for routine monitoring, particularly in settings where no population based electronic health records are available. This highlights the need for more feasible and sustainable data sources and study designs to support ongoing assessment of nirsevimab effectiveness in real-world settings.

The test-negative case–control study design (TND) has been widely used for seasonal influenza vaccine monitoring in the last decades to inform recommendations on target groups and vaccine composition each year [11–13]. It also became a popular method to estimate effectiveness of COVID-19 vaccines after they were rolled out in 2021, and in subsequent years, to evaluate ongoing effectiveness with increasing population immunity and SARS-CoV-2 evolution [14–16]. The particular nature of the TND design, with healthcare-based study recruitment, reduces bias due to healthcare seeking behaviour and permits using it on respiratory infections surveillance data, making it accessible and efficient [12, 17].

To date, there are few examples of TND studies assessing the effectiveness of nirsevimab against RSV hospitalization in the dynamic population of newborns. Existing studies were conducted in settings with low nirsevimab coverage [18, 19] or provided unadjusted estimates, not accounting for calendar time [20] or accounting partially [21].

The objective of this study was to estimate the effectiveness of nirsevimab in preventing RSV-associated hospitalizations during the second season of implementation in Spain. To achieve this, we applied a TND using data from the national acute respiratory infections (ARI) surveillance system, while reflecting on the challenges related to the implementation of the TND in this context.

## Material and methods

### Data sources, participants, and definitions

Within the Spanish ARI surveillance system (SiVIRA), a network of 48 sentinel hospitals covering 14.6 million inhabitants across 16 regions identify all admissions due to acute onset of respiratory symptoms in addition to clinician’s judgement that the symptoms are due to an infection. In children aged under 6 months, admissions due to apnoea or sepsis were also included, following the World Health Organization recommendation [22]. According to the surveillance protocol, all patients fulfilling this case definition admitted on one or two pre-defined weekdays (which vary by hospital, normally Tuesdays and/or Wednesdays) are systematically selected for triple testing of influenza, SARS-CoV-2, and RSV, along with detailed data collection on demographics, clinical status, and vaccination history.

For the purpose of this study, only data from 11 regions with eligible patients and/or at least one patient with reported nirsevimab administration were used. We selected patients born on or after 1 April 2024, hospitalized between the start of the 2024 nirsevimab immunization campaign (regionally variable, between 16 September and 1 October 2024) and 31 March 2025, excluding re-admissions (defined as any admission within 14 days of a previous hospital discharge), and who had been tested by reverse transcription polymerase chain reaction (RT-PCR) and had a valid result for RSV, with the swab taken within 10 days after symptom onset, to ensure adequate sensitivity and specificity of RSV detection. For patients with unknown date of symptom onset or with onset reported on the same day of hospital admission (likely corresponding to a data collection error), imputation was performed by subtracting the median interval between symptom onset and hospitalization estimated among children with complete data (3 days).

Selected patients were classified as cases if they tested positive for RSV or as controls if they tested negative. We restricted the sample of controls to those hospitalized on or after the first hospitalization date of an RSV case in each region, to ensure that they had the opportunity of exposure to the virus.

Cases and controls were classified as immunized with nirsevimab if they had received it up to the day before symptom onset, or as non-immunized if they had never received it up to that date. Children were further stratified into two groups according to the indication for nirsevimab administration: (a) infants born between 1 April 2024 and the start of the immunization campaign, who were eligible for nirsevimab as catch-up immunization and received electronic appointments to attend a public hospital or healthcare centre for nirsevimab administration (catch-up group); and (b) infants born from the start of the immunization campaign until 31 March 2025, who were offered immunization on the first day of life before discharge from hospital (at-birth group). Immunization was free of charge and the recommended dose was 50 mg for those under 5 kg and 100 mg for those weighing ≥5 kg at the time of administration.

Several variables capturing potential risk factors for severe RSV that could also be associated to the likelihood of receiving nirsevimab were collected. Available data on co-morbidities included presence of malformations (e.g., respiratory, circulatory, digestive, and neuromuscular systems), chromosomal abnormalities, including Down syndrome, chronic diseases (cardiovascular, respiratory, liver, and kidney), immunodeficiencies and metabolic disorders. For these, we computed whether ≥1 condition was reported, otherwise not differentiating between reported absence or missing data. Birth weight and gestational age at birth, two continuous variables with a high proportion of missing data, were excluded from the main analysis.

### Statistical analyses

Descriptive analyses were performed. Categorical variables were presented as frequencies and percentages, and quantitative variables were presented as median and interquartile ranges.

The odds of being immunized with nirsevimab were compared between cases and controls using logistic regression. Case–control status was the dependent variable, nirsevimab immunization was the main exposure and the model was adjusted for potential confounders, including week of admission (modelled as natural cubic splines with two inner knots), sex, presence of risk factors for severe RSV (at least 1 condition), age at admission in days, and RSV hospitalization rate (per 100000 population, by region and epidemiological week), both modelled as natural cubic splines with two inner knots (Supplementary Figure S3). The odds ratio (OR) and its 95% confidence interval (95% CI) were estimated, and immunization effectiveness (IE) was derived as IE = (1 − OR) × 100.

The analysis was performed overall and separately for the catch-up and at-birth groups. Within each analysis, we attempted stratification by variables that could present as effect modifiers: time since immunization (0–34, 35–69, and ≥70 days) and age (with different categories for each group: overall and the catch-up group: <60, 60–119, 120–179, and ≥180 days; and for the at-birth group: 0–29, 30–59, 60–119, and ≥120 days). We finally assessed effectiveness separately in the first and the second half of the RSV epidemic (between 1 October 2024 and 5 January 2025 vs. between 6 January and 31 March 2025).

The data used in this study were collected for the purpose of epidemiological surveillance of RSV in Spain and therefore is exempt of acquisition of informed consent from patients and specific ethical clearance.

### Sensitivity analyses

Several sensitivity analyses were conducted to test the impact of study assumptions and modelling choices: (a) Infants who received nirsevimab within 5 days (the median incubation period of RSV) prior to symptom onset were excluded, to avoid including those who might have already been RSV-infected at the time of nirsevimab administration; (b) inclusion of preterm birth (<37 weeks of gestational age) and low birth weight (<2500 g) as covariates in the logistic regression model, restricting to the sub-sample of infants with complete data; (c) exclusion of cases with RSV co-detected with influenza or SARS-CoV-2 and controls with either of those infections.

## Results

### Sample characteristics

**Figure 1.**
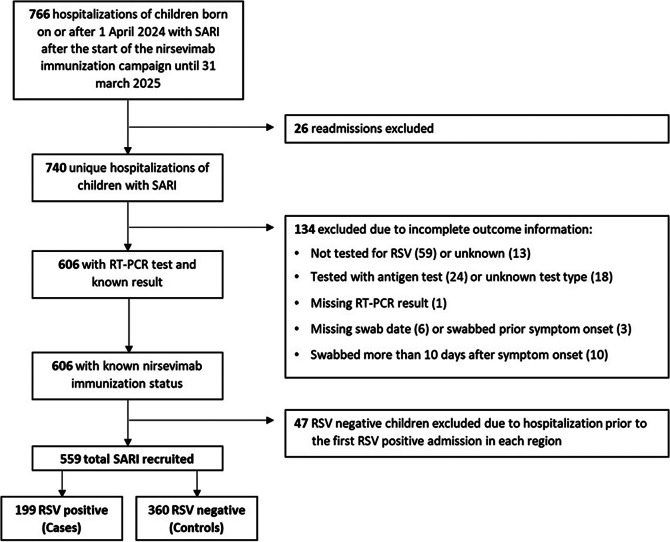
Flow chart of patient selection into the study among those admitted with Severe Acute Respiratory Syndrome (SARI) in hospitals participating in national SARI surveillance.

Of 766 initially eligible children admitted with SARI, 559 (73%) fulfilled the selection criteria and were included in the study, comprising 199 RSV-positive cases and 360 RSV-negative controls (Figure 1). Among them, 102 cases and 147 controls belonged to the catch-up group (67.7% and 84.4% immunized with nirsevimab, respectively) and 97 cases and 213 controls to the at-birth group (70.1% and 87.8% immunized) (Supplementary Figure S1). The distribution of immunization months differed between the two study groups. Among infants in the catch-up group, most received the immunization in September and October, accounting for approximately 26% and 67% of all doses, respectively. In contrast, among infants in the at-birth group, the monthly distribution of immunizations closely mirrored the distribution of births, as these infants were immunized shortly after delivery. The median time from nirsevimab administration to symptom onset was 94 days (interquartile range (IQR): 71–116) in catch-up immunized cases and 99.5 days (IQR: 58–140) in catch-up immunized controls, and 43 days (IQR: 29–66.5) and 40 days (IQR: 20–68) in at-birth immunized cases and controls, respectively (Table 1).

**Table 1.** Characteristics of cases and controls overall and separately in the catch-up and at-birth nirsevimab immunization groups, Spain, 2024/2025 season (based on data from *n* = 559 children)

Characteristic	Overall (*n* = 559)				Catch-up immunization (*n* = 249)								At-birth immunization (*n* = 310)				
	Cases		Controls		Cases					Controls			Cases		Controls		
	*N*		%		*N*		%			*N*	%		*N*	%	*N*	%	
Number of patients	199	35.6	360	64.4	102		41.0			147	59.0		97	31.3	213	68.7	
Sex																	
Male	116	58.3	227	63.1	62		60.8			97	66.0		54	55.7	130	61.0	
Female	83	41.7	133	36.9	40		39.2			50	34.0		43	44.3	83	39.0	
Month of birth																	
April 2024	10	5.0	15	4.2	10		9.8			15	10.2		NA		NA		
May 2024	11	5.5	16	4.4	11		10.8			16	10.9						
June 2024	6	3.0	25	6.9	6		5.9			25	17.0						
July 2024	19	9.6	31	8.6	19		18.6			31	21.1						
August 2024	24	12.1	33	9.2	24		23.5			33	22.5						
September 2024	35	17.6	29	8.1	32		31.4			27	18.4		3	3.1	2	0.9	
October 2024	26	13.1	44	12.2	NA					NA			26	26.8	44	20.7	
November 2024	30	15.1	50	13.9									30	30.9	50	23.5	
December 2024	22	11.1	56	15.6									22	22.7	56	26.3	
January 2025	7	3.5	38	10.6									7	7.2	38	17.8	
February 2025	8	4.2	19	5.3									8	8.3	19	8.9	
March 2025	1	0.5	4	1.1									1	1.0	4	1.9	
Month of admission																	
October 2024	3	1.1	3	0.8	3				2.9	2	1.4		0	0.0	1	0.5	
November 2024	11	5.5	32	8.9	6				5.9	19	12.9		5	5.2	13	6.1	
December 2024	58	29.2	64	17.8	34				33.3	25	17.0		24	24.7	39	18.3	
January 2025	75	37.7	82	22.8	34				33.3	30	20.4		41	42.3	52	24.4	
February 2025	35	17.6	77	21.4	18				17.7	33	22.5		17	17.5	44	20.7	
March 2025	17	8.5	102	28.3	7				6.9	38	25.9		10	10.3	64	30.1	
Age at admission																	
0–29 days	27	13.6	61	16.9	NA					NA			27	27.8	61	28.6	
30–59 days	33	16.6	78	21.7	0		0			2	1.4		33	34.0	76	35.7	
60–89 days	36	18.1	44	12.2	9		8.8			7	4.8		27	27.8	37	17.4	
90–119 days	21	10.6	41	11.4	14		13.7			18	12.2		7	7.2	23	10.8	
120–149 days	21	10.6	28	7.8	19		18.6			15	10.2		2	2.1	13	6.1	
150–179 days	22	11.1	24	6.7	21		20.6			21	14.3		1	1.0	3	1.4	
180–209 days	14	7.0	24	6.7	14		13.7			24	16.3		NA		NA		
210–239 days	11	5.5	21	5.8	11		10.8			21	14.3						
≥240 days	14	7.0	39	10.8	14		13.7			39	26.5						
Previous co-morbidities																	
Malformations	7	3.5	26	7.2		4		3.9		13	8.8		3	3.1	13	6.1	
Cardiovascular disease*	3	1.5	15	4.2		2		2.0		6	4.1		1	1.0	9	4.2	
Respiratory disease*	1	0.5	16	4.4		1		1.0		11	7.5		0	0.0	5	2.4	
Liver disease*	0	0.0	1	0.3		0		0.0		1	0.7		0	0.0	0	0.0	
Kidney disease*	1	0.5	0	0.0		1		1.0		0	0.0		0	0.0	0	0.0	
Immunodeficiencies	2	1.0	3	0.8		1		1.0		1	0.7		1	1.0	2	0.9	
Metabolic disorders	0	0.0	0	0.0		0		0.0		0	0.0		0	0.0	0	0.0	
Gestational age (weeks)																	
≥37	137	68.8	202	56.1		68		66.7		70	47.6		69	71.1	132	62.0	
<37	10	5.0	52	14.4		5		4.9		24	16.3		5	5.2	28	13.2	
Unknown	52	26.1	106	29.4		29		28.4		53	36.1		23	23.7	53	24.9	
Birth weight (g)																	
≥2,500	133	66.8	200	55.6		64		62.8		65	44.2		69	71.1	135	63.4	
<2,500	10	5.0	52	14.4		6		5.9		28	19.1		4	4.1	24	11.3	
Unknown	56	28.1	108	30.0		32		31.4		54	36.7		24	24.7	54	25.4	
RSV viral sub-group																	
A	46	23.1	NA			22		21.6				24		24.7	NA		
B	18	9.1				7		6.9		NA		11		11.3			
Unknown	135	67.8				73		71.6				62		63.9			
Detection of SARS-CoV–2 or influenza																	
SARS-CoV–2	2	1.0	8	2.3		1		1.0		5	3.5	1		1.0	3		1.4
Influenza	4	2.0	29	8.1		2		2.0		16	10.9	2		2.1	13		6.1
No detection	189	95.0	318	88.3		97		95.1		124	84.3	92		94.8	194		91.1
Unknown	4	2.0	5	1.4		2		2.0		2	1.4	2		2.1	3		1.4
Nirsevimab administration																	
No	62	31.2	49	13.6		33		32.4		23	15.7	29		29.9	26	12.2	
Yes	137	68.8	311	86.4		69		67.7		124	84.4	68		70.1	187	87.8	
Immunization month																	
September	19	13.9	34	10.9		18		26.1		33	26.6	1		1.5	1	0.5	
October	67	48.9	112	36.0		48		69.6		82	66.1	19		27.9	30	16.0	
November	27	19.7	57	18.3		2		2.9		5	4.0	25		36.8	52	27.8	
December	15	11.0	54	17.4		1		1.45		3	2.4	14		20.6	51	27.3	
January	4	2.9	30	9.7		0		0.0		1	0.8	4		5.9	29	15.5	
February	4	2.9	20	6.4		0		0.0		0	0.0	4		5.9	20	10.7	
March	1	0.7	4	1.3		0		0.0		0	0.0	1		1.5	4	2.1	
Time since immunization																	
0–34 days	27	19.7	98	31.5		3		4.4		14	11.3	24		35.3	84	44.9	
35–69 days	41	29.9	78	25.1		12		17.4		21	16.9	29		42.6	57	30.5	
≥ 70 days	69	50.4	135	43.4		54		78.3		89	71.8	15		22.1	46	24.6	
Median time (days) since immunization (IQR)	70 (40–101)		56 (30–104)			94 (71–116)				99.5 (58–140)		43 (29–66.5)			40 (20–68)		
Infection severity																	
ICU admission	52	26.1	61	16.9		15		14.7		19	13.0	37		38.1	42	19.7	
Deceased	0	0.0	1	0.3		0		0.0		1	0.7	0		0.0	0	0.0	

ICU, intensive care unit; IQR, interquartile range; NA, not applicable; RSV, respiratory syncytial virus.

* As chronic condition.

**Figure 2.**
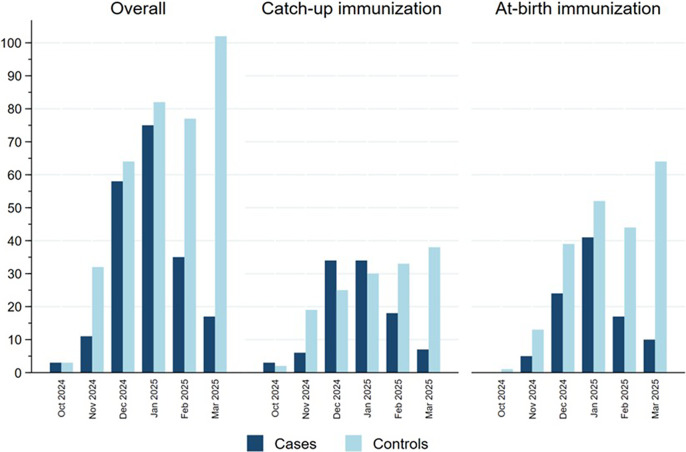
Distribution of cases and controls in the catch-up and at-birth groups by month of hospitalization, Spain, 2024/25 season.

Figure 2 represents the distribution of cases and controls by month of admission, with cases representing the RSV epidemic peaking in January 2025 and controls being more evenly distributed in time, reflecting the distribution of other causes of SARI in this population. Cases were more frequently born between July and September 2024 in the catch-up group and between October and December in the at-birth immunization group corresponding, respectively, to the youngest children born before the RSV epidemic peak (Table 1). Indeed, the distribution of cases and controls by month of admission, age at admission and birthdate were correlated in an age–period–cohort structure, which meant that cases and controls admitted at increasing age tended to be born earlier and belong to a later stage in the RSV epidemic (Supplementary Figure S2).

Compared to controls, cases were less often male, born preterm, of low birth weight, or with underlying co-morbidities (Table 1). Among the 64 cases with available RSV sub-group information, sub-group A was identified in 46 (72%) cases and co-detection of influenza or SARS-CoV-2 was uncommon, observed in only six cases. Other respiratory viruses were not systematically tested nor collected. Intensive care unit admission was recorded for 15 (14.7%) cases in the catch-up group and for 37 (38.1%) cases in the at-birth group, and was lower in controls compared to cases.

### Nirsevimab effectiveness

Effectiveness of nirsevimab in preventing RSV-associated hospitalizations was 65.5% (95% CI: 45.2 to 78.3) overall, 63.6% (95% CI: 26.9 to 81.9) in the catch-up group, and 70.4% (95% CI: 43.4 to 84.5) in the at-birth group (Tables 2 and 3). These adjusted effectiveness estimates did not differ substantially from crude ones, showing low degree of confounding by any of these variables. The additional adjustment for the date of birth did not change results, but was not included since within the age–cohort–period structure it could result in overfitting (Supplementary Table S1).

**Table 2.** Overall effectiveness of nirsevimab immunization against hospitalization for respiratory syncytial virus infection in the first year of life, Spain, 2024/2025 season (based on data from *n* = 559 children)

	Cases		Controls		Crude IE (95% CI)	Adjusted IE** (95% CI)
	Immunized	Total	Immunized	Total		
Overall	137	199	311	360	64.9 (45.0 to 77.6)	65.5 (45.2 to 78.3)
Time since immunization						
0–34 days	27	90*	98	157*	80.1 (63.6 to 89.2)	79.3 (56.0 to 90.2)
35–69 days	41	104*	78	137*	67.1 (42.1 to 81.3)	69.8 (44.0 to 83.7)
≥70 days	69	132*	135	194*	49.9 (16.4 to 70.0)	50.3 (11.3 to 72.1)
Age at admission						
<60 days	38	60	123	139	79.3 (54.5 to 90.6)	80.0 (55.9 to 91.0)
60–119 days	43	57	76	85	67.5 (14.0 to 87.7)	61.0 (–4.1 to 85.4)
120–179 days	31	43	46	52	60.6 (–20.7 to 87.2)	57.2 (–32.8 to 86.2)
≥180 days	25	39	66	84	37.1 (–53.6 to 74.2)	41.8 (–47.0 to 77.0)

CI, confidence intervals; IE, immunization effectiveness.

* The number of total cases and controls in time since immunization intervals include the total number of non-immunized infants plus the number in each time since immunization interval, respectively, for cases and controls.

** Logistic regression, adjusted by sex, risk factors, week of admission (NCS with two inner knots), age at admission (NCS with two inner knots), and RSV hospitalization rate (per 100000 population, by region and epidemiological week) (NCS with two inner knots). All models are based on ≥10 cases per variable.

**Table 3.** Effectiveness of catch-up and at-birth nirsevimab immunization against hospitalization for respiratory syncytial virus infection in the first year of life, Spain, 2024/2025 season (based on data from *n* = 559 children)

	Cases		Controls		Crude IE (95% CI)	Adjusted IE** (95% CI)
	Immunized	Total	Immunized	Total		
Catch-up immunization						
Overall	69	102	124	147	58.8 (21.3 to 78.5)	63.6 (26.9 to 81.9)
Time since immunization						
0–34 days	3	37*	14	37*	NA	NA
35–69 days	12	46*	21	44*	74.0 (30.9 to 90.2)	80.0 (42.2 to 93.1)
≥70 days	54	88*	89	112*	47.0 (–6.7 to 73.7)	53.6 (1.7 to 78.1)
Age at admission						
<60 days	0	0	2	2	NA	NA
60–119 days	16	23	23	25	83.3 (3.8 to 97.1)	81.1 (–27.7 to 97.2)
120–179 days	28	40	33	36	77.2 (8.3 to 94.3)	74.8 (–4.8 to 93.9)
≥180 days	25	39	66	84	38.2 (–51.0 to 74.7)	43.9 (–43.2 to 78.0)
At-birth immunization						
Overall	68	97	187	213	68.4 (40.6 to 83.2)	70.4 (43.4 to 84.5)
Time since immunization						
0–34 days	24	53*	84	111*	77.6 (52.4 to 89.5)	82.2 (57.1 to 92.6)
35–69 days	29	58*	57	84*	62.4 (21.7 to 82.0)	65.8 (22.9 to 84.8)
≥70 days	15	44*	46	73*	58.3 (3.3 to 82.1)	35.2 (–105 to 79.6)
Age at admission						
0–29 days	16	27	54	61	81.7 (41.0 to 94.3)	89.2 (59.0 to 97.2)
30–59 days	22	33	67	76	76.3 (31.9 to 91.7)	80.3 (41.1 to 93.4)
60–89 days	21	27	32	37	61.0 (–61.2 to 90.5)	51.9 (–106 to 88.8)
≥90 days	9	10	34	39	NA	NA

CI, confidence intervals; NA, not applicable; IE, immunization effectiveness.

* The number of total cases and controls in time since immunization intervals include the total number of non-immunized infants plus the number in each time since immunization interval, respectively, for cases and controls.

** Logistic regression, adjusted by sex, risk factors, week of admission (NCS with two inner knots), age at admission (NCS with two inner knots), and RSV hospitalization rate (per 100000 population, by region and epidemiological week) (NCS with two inner knots). All models are based on between 5 and 10 cases per variable.

By time since immunization, a decreasing gradient was found overall, as well as for the catch-up and at-birth groups analysed separately (Tables 2 and 3). Overall immunization effectiveness was estimated at 79.3% (95% CI: 56.0 to 90.2) in the first 0–34 days, declining to 69.8% (44.0 to 83.7) at 35–69 days and 50.3% (11.3 to 72.1) from 70 days onwards. This progressive decline in effectiveness was accompanied by a widening of confidence intervals.

Age-stratified analyses similarly showed a decline in effectiveness with increasing age, mainly in the catch-up immunization group, albeit with wide confidence intervals that precluded strong conclusions on heterogeneity of the effect by age.

Finally, effectiveness in the first half of the RSV epidemic was higher for all groups, of 80.5% (55.5 to 81.5) overall, 86.3% (50.9 to 96.2) for catch-up, and 79.6% (26.9 to 94.3) for at-birth immunization, compared to effectiveness found for the second half, which was 53.6% (15.7 to 74.4) overall, 41.0% (–52.3 to 77.1) for catch-up, and 63.9% (19.0 to 83.9) for at-birth immunization (Supplementary Table S2).

### Results of sensitivity analyses

When restricting the analysis to infants with available data on preterm birth and low birth weight (Supplementary Table S3), a total of 393 children remained in the analysis (70.3% of the original 559). The catch-up group included 70 cases (68.6% of 102) and 93 controls (63.3% of 147), while the at-birth group included 72 cases (74.2% of 97) and 158 controls (74.2% of 213). After adjusting for these covariates, effectiveness in the catch-up group decreased to 56.1%, compared with 63.6% before the exclusion, whereas effectiveness in the at-birth group increased slightly to 73.4% (from 70.4%).

Excluding cases and controls with influenza or SARS-CoV-2, 516 children remained available for analysis (92.3%). The catch-up group included 99 cases (97.1%) and 126 controls (85.7%), while the at-birth group comprised 94 cases (96.9%) and 197 controls (92.5%). Effectiveness increased to 67.4% in the catch-up group and to 73.1% in the at-birth group.

Finally, excluding infants who received nirsevimab within 5 days of symptom onset, 552 children remained available for analysis (98.7%). All cases and controls in the catch-up group were retained, while in the at-birth group, seven controls were excluded. Effectiveness in the catch-up group remained at 63.6%, and in the at-birth group was 69.5%.

## Discussion

In this test-negative case–control study conducted during the 2024/2025 RSV season in Spain, nirsevimab demonstrated an overall effectiveness of 65.5% (95% CI: 45.2 to 78.3) in preventing RSV-associated hospitalizations among infants under 1 year of age. Effectiveness was estimated at 63.6% (95% CI: 26.9 to 81.9) in the catch-up group and 70.4% (95% CI: 43.4 to 84.5) in the at-birth group. Confidence intervals were wide, particularly when attempting sub-group stratification, mainly owing to the low number of unvaccinated controls, as nirsevimab coverage in Spain was around 90% [4].

These effectiveness estimates are at the lower end of those reported by observational studies during the previous season, ranging from 70% to 90% [5–7, 18, 20, 23–25]. The wide variability in effectiveness estimates may be partly explained by differences in study design (including TND, screening, cohorts, both retrospective and prospective, and population based matched case–control approaches), geographic scope (regional vs. national) and immunization strategies (catch-up, at-birth, or both). Moreover, some studies did not rely exclusively on PCR for RSV confirmation or did not capture hospitalizations occurring throughout the entire immunization period (up to 31 March 2024), restricting to the early RSV epidemic. The ongoing effectiveness of nirsevimab in the second implementation season is further supported by the similar incidence of RSV in Spain among children under 1 year in the 2023–2024 and 2024–2025 seasons [26].

Our study found a gradient by time since immunization, albeit with wide confidence intervals. A TND study from the United States, with much lower nirsevimab coverage among target population, also found that effectiveness against RSV-associated hospitalizations declined gradually from 91% (95% CI: 71 to 98) at 2 weeks post-immunization to 74% (95% CI: 24 to 92) at 10 weeks, and 49% (95% CI: –149 to 88) after 16 weeks, though the analysis imposed a monotonic structure on the regression coefficients [19], meaning that effectiveness was constrained to decline over time, influencing the magnitude and trajectory of the decline. On the other hand, recent data from the HARMONIE phase IIIb trial [27], conducted across several European countries under conditions closely resembling real-world clinical practice, demonstrated an overall effectiveness of 83% within 180 days of nirsevimab administration against RSV-related hospitalizations in infants during the 2022/2023 season.

While a decline in effectiveness is expected beyond 10 weeks, consistent with nirsevimab’s half-life, our results likely overestimate the magnitude of this decrease. As the RSV season progresses, the likelihood of infection increases among susceptible individuals, especially those who are non-immunized. This dynamic introduces a bias known as differential depletion of susceptibles, which creates an increasing imbalance in susceptibility between the immunized and non-immunized groups over time. The extent of this imbalance grows with higher vaccine effectiveness and infection attack rates, ultimately resulting in an underestimation of the actual benefit of immunization [28]. This bias may be particularly relevant for RSV (e.g., as compared to influenza), given its very high incidence. In Europe, the cumulative attack rate for medically attended RSV infections among healthy term infants in the first year of life has been estimated at 14.1%, rising to 25% under active surveillance [2]. Yet this may even underrepresent the true burden of disease, since other studies estimate that most children have acquired RSV by the age of 2 [29]. Accounting for prior infection might mitigate this bias, but this variable could not be collected in our study and, moreover, most patients with RSV infection are not tested in routine practice, precluding valid identification of previous infections.

A combined effect of true waning of the nirsevimab protection and the previously described bias by differential depletion of susceptibles could also explain why effectiveness in our study was much lower in the second part of the respiratory virus season in the catch-up group, who were mostly immunized in October, while it was more preserved in the at-birth immunization group, who dynamically entered the population at risk and were immunized along the period. This highlights that in the presence of time-varying effectiveness, the duration of the study will influence the overall effectiveness, particularly for catch-up groups.

Due to the interplay between progressively decreasing maternal antibodies and increasing development of the respiratory and immune systems along the first months of life [30, 31], it is possible that the effectiveness of nirsevimab varies by age. In our study, there was an age-related gradient in effectiveness, mainly in the catch-up immunization group, with highest estimates (80–82%) observed among the youngest children, that gradually declined in older age groups to reach the null, though with very wide confidence intervals that preclude interpretation. Moreover, due to the high collinearity between age and time since immunization, particularly in the group targeted for catch-up, age gradients are difficult to disentangle from those of time since immunization. Also, age–period–cohort structures determine that infants admitted at an older age are those at the end of the RSV epidemic, thus risking the highest degree of the above-mentioned potential bias due to differential depletion of susceptibles [28, 32]. These limitations curtail our capacity to study potential age-related differences in protection.

The TND was developed as a practical alternative to cohort studies, aiming to adjust for differences in healthcare-seeking behaviour between vaccinated and unvaccinated individuals by restricting selection to those who seek medical care [12, 17, 33]. Because of its demonstrated validity under appropriate conditions, as well as its high efficiency and cost-effectiveness for the evaluation of a range of outcomes, the TND is often used for observational vaccine effectiveness evaluation when appropriate. However, its validity depends on the assumed underlying data-generating structure, the factors influencing the selection of cases and controls into the study and the assessment of their vaccination status. Moreover, the amount and quality of information available to the researchers, along with other factors [34–37], create unique challenges for different viruses, target populations, and epidemiological contexts.

It is plausible that core validity assumptions of TND [11, 12] are met in our study. Residual confounding by healthcare seeking behaviour despite restricting to those who seek care in the TND is possible [12], but unlikely, since in a national health system like the one in Spain we can assume that any patient in need of hospital admission was admitted and available for selection into the study regardless of case severity. It is also plausible that administration of nirsevimab did not influence the risk of severe respiratory infections caused by pathogens other than RSV. Additionally, although maternal vaccination against influenza and SARS-CoV-2, and vaccination against influenza for infants older than 6 months, are offered in Spain, exclusion of cases and controls with laboratory-confirmed influenza and SARS-CoV-2 did not substantially change our estimates.

Other strengths of this study include the use of a nationally representative sample of children, with a uniform case definition applied across hospitals and regions. Systematic testing of admitted patients, conducted on one or two pre-defined weekdays each week, depending on the hospital, helped minimize selection bias associated with symptom-based testing, and possibly minimized the heterogeneity due to different admission and testing criteria across hospitals [28, 34]. Exclusive reliance on RT-PCR performed within 10 days of symptom onset for RSV confirmation enhances sensitivity and specificity reducing misclassification of the case versus control status [38]. To ensure temporal comparability between cases and controls, controls admitted during the weeks preceding the first laboratory-confirmed RSV case in each region were excluded from the analysis; this approach minimized the inclusion of controls admitted outside the circulation period of RSV, reducing potential bias due to differential exposure risk and improving the accuracy of vaccine effectiveness estimates [11].

On the other hand, several limitations may have influenced the validity and precision of our results. First, although SiVIRA gathers data from 48 sentinel hospitals, only patients admitted on specific weekdays are included, which limits the available sample size and may affect representativeness. Second, well-established host risk factors for RSV hospitalization, such as low birth weight and prematurity, were poorly captured and could not be accounted for. Co-morbidities also showed a high number of missing values, raising concerns that adjustment by this variable may have been insufficient. Maternal RSV vaccination could not be collected, possibly biasing effectiveness downwards by decreasing the risk in the non-immunized. Other factors potentially associated with RSV risk and nirsevimab acceptance, such as number of siblings, household crowding, breastfeeding, toxic exposures such as tobacco use during pregnancy, day-care exposure, and socio-economic status were not collected, with possible residual confounding. Finally, we cannot rule out misclassification of the outcome and/or the exposure. We did not collect the type of swab, possibly being nasal and/or oropharyngeal in these very young children, instead of the most sensitive nasopharyngeal sample [39]. Even if test specificity is more relevant than sensitivity for validity of TND estimates, low sensitivity can also result in bias towards the null when immunization coverage is high, like in our setting [17, 38, 40]. Regarding the immunization status, it was collected by the attending physicians, public health officers or by cross-matching with vaccination registries, depending on the region. Since in Spain there is no centralized vaccination registry, quality assessments of the collected information could not be performed.

## Conclusions

Nirsevimab effectiveness against hospitalization due to RSV estimated using TND with national surveillance data in children immunized during the 2024/2025 season was in the lower end of the range of estimates from the previous season, when nirsevimab was first rolled out in Spain. The high immunization coverage increased the sample size requirement, resulting in wide confidence intervals that precluded strong conclusions on sub-group analyses, though gradients by time since immunization and age were suggested. This, coupled with the low frequency of RSV hospitalization in the population targeted for nirsevimab makes the TND the most feasible approach for onwards monitoring and calls for collaborative studies to increase study power and advance research into challenges associated with the implementation of TND in the dynamic population of newborn children.

## Supporting information

10.1017/S0950268825100782.sm001 Campos Mena et al. supplementary material Campos Mena et al. supplementary material

## Data Availability

All relevant data are provided in the tables within the main manuscript and the Supplementary Material.

## Supplementary material

The supplementary material for this article can be found at http://doi.org/10.1017/S0950268825100782.
