# Supplementary material for: Monitoring effectiveness of nirsevimab immunization against RSV hospitalization using surveillance data: a test-negative case–control study, Spain, October 2024–March 2025
Source: Epidemiol Infect. 2025 Dec 9;154:e5. doi: 10.1017/S0950268825100782 (PMC12780921; doi:10.1017/S0950268825100782)
Supplement: Campos Mena et al. supplementary material [file S0950268825100782sup001.docx]

**Figure S1. Flow chart of patient selection into the study among infants admitted with Severe Acute Respiratory Infection (SARI) in hospitals participating in national SARI surveillance, in the catch-up and at-birth nirsevimab immunization groups**

1. **
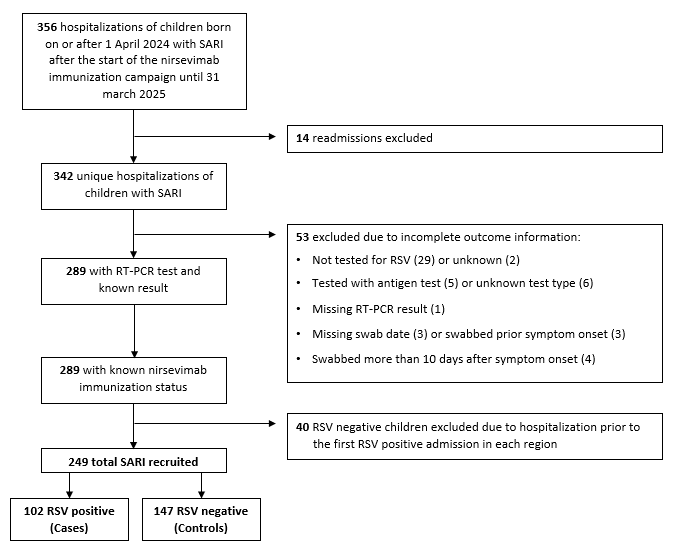
**Catch-up group
2. At-birth group

**
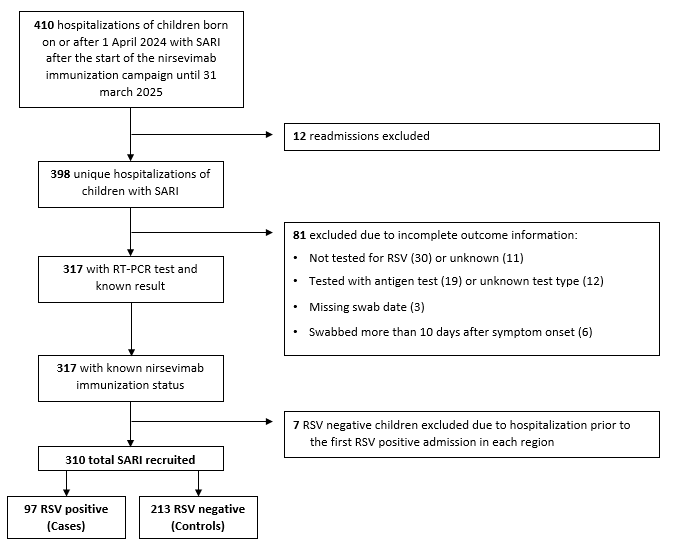
**

**Figure S2. Distribution of cases and controls by age at admission (coloured categories) in the catch-up and at-birth nirsevimab immunization groups, according to (a) month of birth in the x-axis or (b) month of hospitalization in the x-axis, Spain, 2024/25 season.**

1. Month of birth


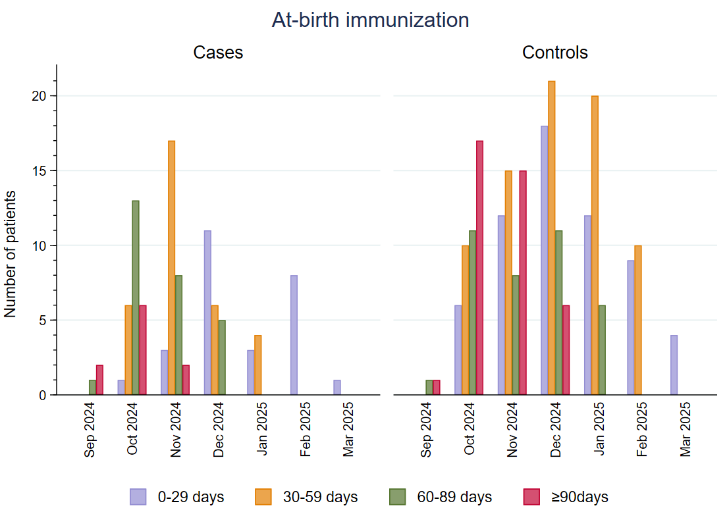

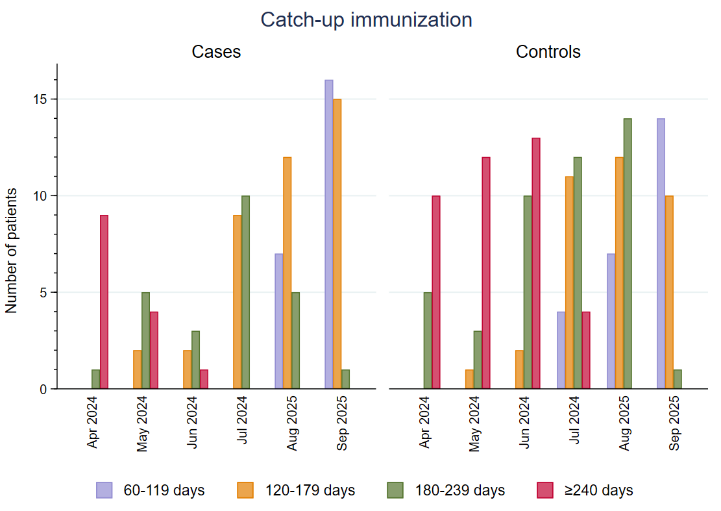


1.
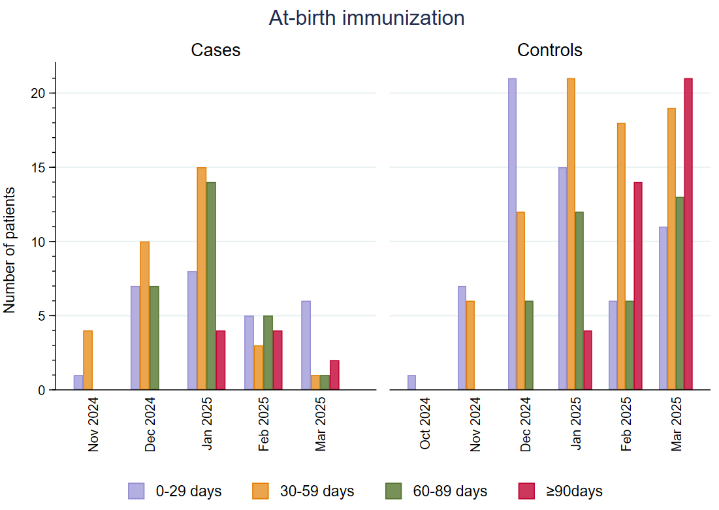
**
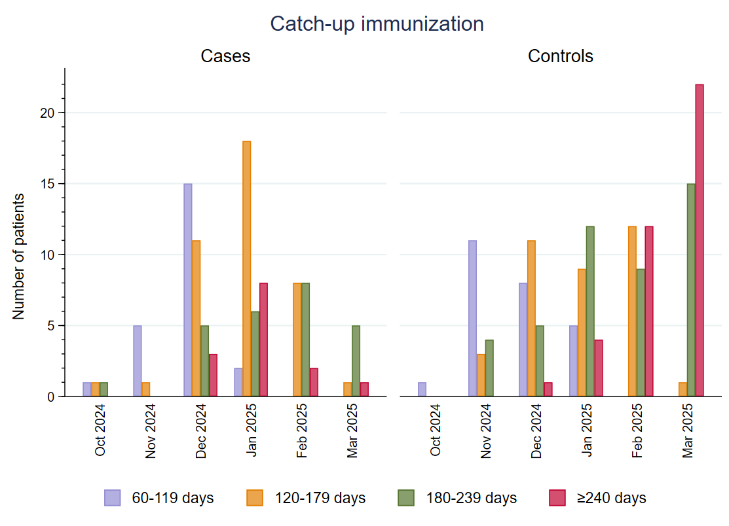
**Month of hospitalization

**Figure S3. Estimation of the crude effects of epidemiological week of hospital admission, age in days and RSV hospitalization rate (per 100,000 population, by region and epidemiological week) —each modelled using a natural cubic spline with 2 inner knots— on the probability of being a case.**


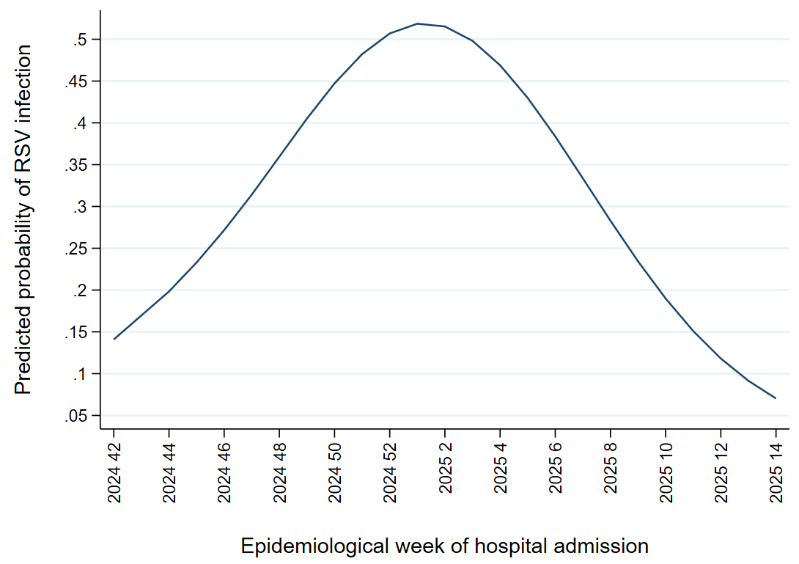

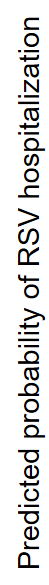

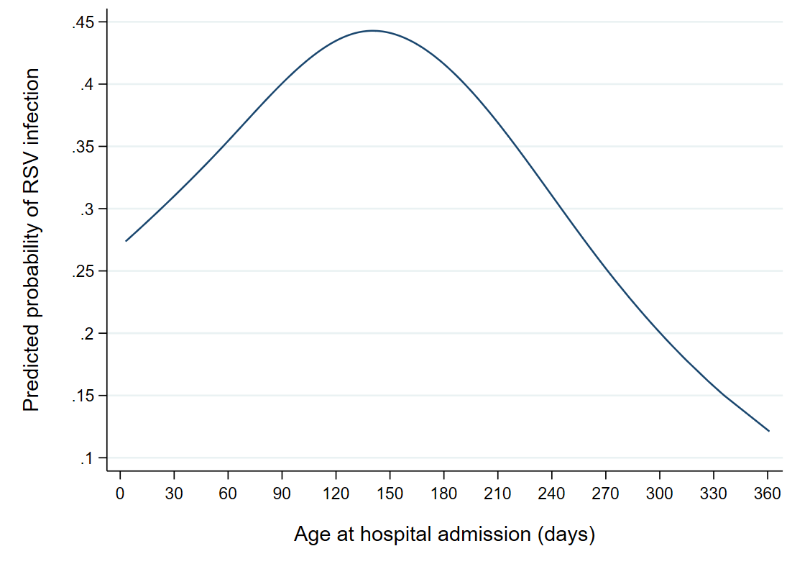

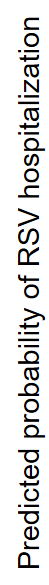


**
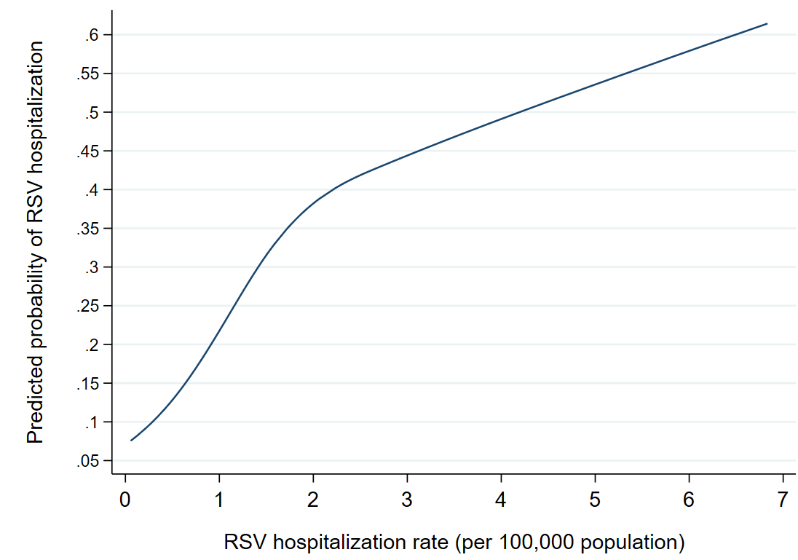
**

**Table S1. Estimated effectiveness (95% Confidence Intervals) of nirsevimab on RSV hospital admissions with or without adjustment by** **epidemiological week of admission (referred to as week), age at admission (in days) and birthdate, modelled as natural cubic splines with 2 inner knots. All models are adjusted by sex, presence of previous comorbidities and RSV hospitalization rate (per 100,000 population, by region and epidemiological week) modelled as natural cubic splines with 2 inner knots.**

|  | **Adjusted by week** | | **Adjusted by age** | | **Adjusted by birth date** | | **Adjusted by week and age** | | **Adjusted by week and birthdate** | | **Adjusted by 1, 2 and 3** |
| --- | --- | --- | --- | --- | --- | --- | --- | --- | --- | --- | --- |
| **Overall** | 64.6 (44.2 ; 77.6) | | 65.8  (46.0 ; 78.4) | | 65.9 (46.0 ; 78.5) | | 65.5  (45.2 ; 78.3) | | 65.2  (44.5 ; 78.1) | | 65.1  (44.4 ; 78.1) |
| **Time since immunization** | |  | |  | |  | |  | |  | |
| 0-34 days | 80.3  (63.5 ; 89.4) | | 75.1  (48.9 ; 87.9) | | 74.4 (51.6 ; 86.4) | | 79.3 (56.0 ; 90.2) | | 81.4 (63.0 ; 90.7) | | 78.7  (55.5 ; 89.8) |
| 35-69 days | 67.9  (43.1 ; 81.9) | | 68.7  (42.3 ; 83.0) | | 65.7 (38.7 ; 80.8) | | 69.8 (44.0 ; 83.7) | | 67.5 (41.4 ; 82.0) | | 69.9  (44.3 ; 83.8) |
| ≥70 days | 48.4 (13.1 ; 69.3) | | 57.3  (25.6 ; 75.5) | | 60.3 (32.4 ; 76.7) | | 50.3 (11.3 ; 72.1) | | 46.0 (2.4 ; 70.0) | | 47.4  (4.3 ; 71.1) |
| **Age at admission** | | |  | |  | |  | |  | |  |
| <60 days | 79.9  (55.6 ; 90.9) | | 79.1 (54.4 ; 90.4) | | 79.3 (54.6 ; 90.5) | | 80.0 (55.9 ; 91.0) | | 78.8 (53.5 ; 90.4) | | 78.9  (53.9 ; 90.4) |
| 60–119 days | 62.5  (0.6 ; 85.9) | | 61.6 (-1.8 ; 85.5) | | 63.2 (2.7 ; 86.1) | | 61.0 (-4.1 ; 85.4) | | 62.1 (-1.3 ; 85.8) | | 60.0  (-8.1 ; 85.2) |
| 120–179 days | 57.1 (-34.3 ; 86.3) | | 58.8 (-29.5 ; 86.9) | | 58.3 (-34.7 ; 87.1) | | 57.2 (-32.8 ; 86.2) | | 57.6 (-35.5 ; 86.7) | | 57.6  (-35.4 ; 86.7) |
| ≥180 days | 42.8 (-44.1 ; 77.3) | | 46.1 (-34.6 ; 78.4) | | 47.7 (-29.6 ; 78.9) | | 41.8 (-47.0 ; 77.0) | | 42.1 (-46.6 ; 77.1) | | 42.3  (-46.6 ; 77.3) |

**Table S2. Overall, catch-up and at-birth effectiveness of nirsevimab immunization against hospitalization for respiratory syncytial virus infection in the first year of life, by calendar period of admission, Spain, 2024/25 season.**

1. **Hospitalizations between 01/10/2024 and 05/01/2025**

|  | **Cases** | | **Controls** | | **Crude IE (95% CI)** | **Adjusted IE****† **(95% CI)** |
| --- | --- | --- | --- | --- | --- | --- |
|  | **Immunized** | **Total** | **Immunized** | **Total** |  |  |
| **Overall** | 56 | 86 | 104 | 116 | 78.8 (54.6 ; 90.1) | 80.5 (55.5 ; 81.5) |
| **Time since immunization** |  |  |  |  |  |  |
| 0-34 days | 17 | 47* | 54 | 66* | 86.2 (66.8 ; 94.3) | 82.6 (50.0 ; 93.9) |
| 35-69 days | 22 | 52* | 34 | 46* | 73.8 (37.2 ; 89.1) | 82.5 (52.9 ; 93.5) |
| ≥70 days | 17 | 47* | 16 | 28* | 65.8 (7.7 ; 87.3) | 72.1 (11.9 ; 91.1) |
| **Catch-up immunization** | | | | | | |
| **Overall** | 28 | 47 | 47 | 53 | 84.4 (52.1 ; 94.9) | 86.3 (50.9 ; 96.2) |
| **Time since immunization** |  |  |  |  |  |  |
| 0-34 days | 2 | 21* | 13 | 19* | NA | NA |
| 35-69 days | 12 | 31* | 20 | 26* | 81.3 (33.2 ; 94.8) | 85.9 (38.3 ; 96.8) |
| ≥70 days | 14 | 33* | 14 | 20* | 79.7 (22.1 ; 94.7) | 79.1 (8.8 ; 95.2) |
| **At-birth immunization** | | | | | | |
| **Overall** | 28 | 39 | 57 | 63 | 71.3 (12.3 ; 90.6) | 79.6 (26.9 ; 94.3) |
| **Time since immunization** |  |  |  |  |  |  |
| 0-34 days | 15 | 26* | 41 | 47* | 79.0 (30.0 ; 93.7) | 84.4 (33.4 ; 96.3) |
| 35-69 days | 10 | 21* | 14 | 20* | 60.5 (-46.9 ; 89.4) | 70.5 (-70.5; 94.9) |
| ≥70 days | 3 | 14* | 2 | 8* | NA | NA |

1. **Hospitalizations between 0/01/2025 and 31/03/2025**

|  | **Cases** | | **Controls** | | **Crude IE (95% CI)** | **Adjusted IE**** † **(95% CI)** |
| --- | --- | --- | --- | --- | --- | --- |
|  | **Immunized** | **Total** | **Immunized** | **Total** |  |  |
| **Overall** | 81 | 113 | 207 | 244 | 53.3 (16.8 ; 73.8) | 53.6 (15.7 ; 74.4) |
| **Time since immunization** |  |  |  |  |  |  |
| 0-34 days | 10 | 42* | 44 | 81* | 72.0 (32.5 ; 88.4) | 78.3 (32.4 ; 93.1) |
| 35-69 days | 19 | 51* | 44 | 81* | 61.0 (15.7 ; 82.0) | 54.6 (-9.8 ; 81.2) |
| ≥70 days | 52 | 84* | 119 | 156* | 42.2 (-7.4 ; 68.9) | 39.0 (-25.4 ; 70.3) |
| **Catch-up immunization** | | | | | | |
| **Overall** | 41 | 55 | 77 | 94 | 23.1 (-83.3 ; 67.8) | 41.0 (-52.3 ; 77.1) |
| **Time since immunization** |  |  |  |  | NA | NA |
| 0-34 days | 1 | 15* | 1 | 18* |  |  |
| 35-69 days | 0 | 14* | 1 | 18* |  |  |
| ≥70 days | 40 | 54* | 75 | 92* |  |  |
| **At-birth immunization** | | | | | | |
| **Overall** | 40 | 58 | 130 | 150 | 66.4 (26.5 ; 84.7) | 63.9 (19.0 ; 83.9) |
| **Time since immunization** |  |  |  |  |  |  |
| 0-34 days | 9 | 27* | 43 | 63* | NA | NA |
| 35-69 days | 19 | 37* | 43 | 63* | 62.0 (5.4 ; 84.8) | 57.7 (-49.1 ; 81.7) |
| ≥70 days | 12 | 30* | 44 | 64* | 60.9 (-2.4 ; 85.1) | 32.7 (-155 ; 82.3) |

IE: Immunization Effectiveness; CI: confidence intervals.

* The number of total cases and controls in time since immunization intervals include the total number of non-immunized infants plus the number in each time since immunization interval, respectively, for cases and controls.

**Logistic regression, adjusted by sex, risk factors, week of admission (NCS with 2 inner knots), age at admission (NCS with 2 inner knots) and RSV hospitalization rate (per 100,000 population, by region and epidemiological week) (NCS with 2 inner knots).

†Estimate based on events-per-variable ratio between 5 and 10.

**Table S3. Results from sensitivity analyses**

1. Inclusion of preterm birth (<37 weeks of gestational age) and low birth weight (<2,500 g) as covariates in the logistic regression model, restricting to the subsample of infants with complete data (N = 393 children).

|  | **Cases** | | **Controls** | | **Crude IE (95% CI)** | **Adjusted IE****  **(95% CI)** |  |
| --- | --- | --- | --- | --- | --- | --- | --- |
|  | **Immunized** | **Total** | **Immunized** | **Total** |  |  |  |
| **Overall** | 106 | 142 | 221 | 251 | 61.0 (31.4 ; 77.9) | 65.5 (36.5 ; 81.2) |  |
| **Time since immunization** |  |  |  |  |  |  |  |
| 0-34 days | 23 | 59* | 72 | 102* | 75.7 (50.2 ; 88.1) | 67.8 (21.6 ; 86.7) |  |
| 35-69 days | 31 | 67* | 61 | 91* | 66.2 (32.8 ; 83.0) | 71.4 (37.2 ; 86.9) |  |
| ≥70 days | 52 | 88* | 88 | 118* | 41.4 (-11.4 ; 69.2) | 59.5 (13.2 ; 81.1) |  |
| **Age at admission** |  |  |  |  |  |  |  |
| <60 days | 31 | 43 | 99 | 109 | 77.3 (38.5 ; 91.6) | 79.5 (43.1 ; 92.6)† |  |
| 60–119 days | 31 | 39 | 52 | 59 | 56.6 (-40.1 ; 86.5) | 53.8 (-60.9 ; 86.7)† |  |
| 120–179 days | 21 | 27 | 32 | 36 | 53.0 (-95.8 ; 88.7) | 54.9 (-100 ; 89.9)† |  |
| ≥180 days | 23 | 33 | 38 | 47 | 23.3 (-136 ; 75.1) | 40.7 (-101 ; 82.5)† |  |
| **Catch-up immunization** | | | | | | | |
| **Overall** | 51 | 70 | 80 | 93 | 53.9 (-6.5 ; 80.1) | 56.1 (-10.6 ; 82.6)† |  |
| **Time since immunization** |  |  |  |  |  |  |  |
| 0-34 days | 2 | 21* | 10 | 23* | NA | NA |  |
| 35-69 days | 9 | 28* | 14 | 27* | 69.8 (0.3 ; 90.8) | 68.6 (-19.4 ; 91.8)† |  |
| ≥70 days | 40 | 59* | 56 | 69* | 39.8 (-48.4 ; 75.5) | 46.9 (-42.2 ; 80.2)† |  |
| **Age at admission** |  |  |  |  |  |  |  |
| <60 days | 0 | 0 | 2 | 2 | NA | NA |  |
| 60–119 days | 10 | 13 | 16 | 18 | 68.6 (-147 ; 96.0) | 62.2 (-296 ; 96.4)† |  |
| 120–179 days | 18 | 24 | 24 | 26 | 74.2 (-46.0 ; 95.5) | 74.7 (-63.2 ; 96.1)† |  |
| ≥180 days | 23 | 33 | 38 | 47 | 19.6 (-156 ; 74.7) | 31.8 (-138 ; 80.5)† |  |
| **At-birth immunization** | | | | | | | |
| **Overall** | 55 | 72 | 141 | 158 | 64.3 (22.0 ; 87.7) | 73.4 (37.8 ; 88.6)† |  |
| **Time since immunization** |  |  |  |  |  |  |  |
| 0-34 days | 21 | 38* | 62 | 79* | 71.1 (29.5 ; 88.2) | 75.0 (25.4 ; 91.6)† |  |
| 35-69 days | 22 | 39* | 47 | 64* | 63.2 (10.0 ; 85.0) | 76.3 (32.5 ; 91.7)† |  |
| ≥70 days | 12 | 29* | 32 | 49* | 50.4 (-36.7 ; 82.0) | 64.1 (-51.8 ; 91.5)† |  |
| **Age at admission** |  |  |  |  |  |  |  |
| 0–29 days | 13 | 19 | 43 | 48 | 75.6 (-1.3 ; 94.1) | 91.8 (54.5 ; 98.5)† |  |
| 30–59 days | 18 | 24 | 54 | 59 | 78.1 (12.4 ; 94.5) | 81.9 (21.2 ; 95.9)† |  |
| 60–89 days | 17 | 21 | 20 | 24 | 36.1 (-225 ; 87.4) | 33.5 (-310 ; 89.2)† |  |
| ≥90 days | 7 | 8 | 24 | 27 | NA | NA |  |

IE: Immunization Effectiveness; CI: confidence intervals; NA: not applicable.

* The number of total cases and controls in time since immunization intervals include the total number of non-immunized infants plus the number in each time since immunization interval, respectively, for cases and controls.

**Logistic regression, adjusted by sex, risk factors, week of admission (NCS with 2 inner knots), age at admission (NCS with 2 inner knots) and RSV hospitalization rate (per 100,000 population, by region and epidemiological week) (NCS with 2 inner knots).

†Estimate based on events-per-variable ratio between 5 and 10.

1. Exclusion of cases with RSV co-detected with influenza or SARS-CoV-2 and controls with either of those infections (N = 516 children)

|  | **Cases** | | **Controls** | | **Crude IE (95% CI)** | **Adjusted IE****  **(95% CI)** |  |
| --- | --- | --- | --- | --- | --- | --- | --- |
|  | **Immunized** | **Total** | **Immunized** | **Total** |  |  |  |
| **Overall** | 134 | 193 | 283 | 323 | 68.7 (49.1 ; 80.8) | 69.8 (50.1 ; 81.7) |  |
| **Time since immunization** |  |  |  |  |  |  |  |
| 0-34 days | 27 | 87* | 92 | 133* | 82.5 (66.8 ; 90.7) | 81.3 (59.5 ; 91.4) |  |
| 35-69 days | 40 | 100* | 73 | 114* | 72.8 (50.2 ; 85.1) | 74.0 (50.0 ; 86.4) |  |
| ≥70 days | 67 | 127* | 118 | 159* | 51.5 (15.5 ; 72.2) | 53.8 (12.6 ; 75.6) |  |
| **Age at admission** |  |  |  |  |  |  |  |
| <60 days | 38 | 60 | 116 | 131 | 80.4 (55.6 ; 91.3) | 81.5 (57.9 ; 91.9) |  |
| 60–119 days | 41 | 55 | 68 | 76 | 71.7 (20.5 ; 89.9) | 66.7 (5.5 ; 88.2) |  |
| 120–179 days | 30 | 41 | 43 | 48 | 65.5 (-16.7 ; 89.8) | 62.1 (-29.5 ; 88.9) |  |
| ≥180 days | 25 | 37 | 56 | 68 | 36.2 (-78.1 ; 77.1) | 40.8 (-74.1 ; 79.9) |  |
| **Catch-up immunization** | | | | | | | |
| **Overall** | 69 | 99 | 110 | 126 | 65.0 (26.3 ; 83.4) | 67.4 (28.5 ; 85.1)† |  |
| **Time since immunization** |  |  |  |  |  |  |  |
| 0-34 days | 3 | 34* | 13 | 29* | NA | NA |  |
| 35-69 days | 12 | 43* | 19 | 35* | 79.5 (41.8 ; 92.8) | 82.2 (45.4 ; 94.2)† |  |
| ≥70 days | 54 | 85* | 78 | 94* | 51.6 (-8.9 ; 78.5) | 55.7 (-4.3 ; 81.2)† |  |
| **Age at admission** |  |  |  |  |  |  |  |
| <60 days | 0 | 0 | 2 | 2 | NA | NA |  |
| 60–119 days | 16 | 23 | 21 | 23 | 82.4 (-4.1 ; 97.0) | 82.4 (-22.3 : 97.5)† |  |
| 120–179 days | 28 | 39 | 31 | 33 | 82.7 (11.2 ; 96.6) | 80.6 (-2.6 ; 96.3)† |  |
| ≥180 days | 25 | 37 | 56 | 68 | 37.0 (-78.8 ; 77.8) | 41.8 (-72.1 ; 80.3)† |  |
| **At-birth immunization** | | | | | | | |
| **Overall** | 65 | 94 | 173 | 197 | 71.4 (44.6 ; 85.2) | 73.1 (47.0 ; 86.4)† |  |
| **Time since immunization** |  |  |  |  |  |  |  |
| 0-34 days | 24 | 53* | 79 | 104* | 79.1 (54.5 ; 90.4) | 83.4 (58.9 ; 93.3)† |  |
| 35-69 days | 28 | 57* | 54 | 79* | 66.7 (28.4 ; 84.5) | 71.4 (33.0 ; 87.8)† |  |
| ≥70 days | 13 | 42* | 40 | 65* | 61.7 (6.3 ; 84.4) | 27.7 (-155 ; 79.5)† |  |
| **Age at admission** |  |  |  |  |  |  |  |
| 0–29 days | 16 | 27 | 49 | 55 | 83.4 (42.7 ; 95.2) | 92.3 (66.7 ; 98.2)† |  |
| 30–59 days | 22 | 33 | 65 | 74 | 75.8 (29.9 ; 91.7) | 79.0 (37.3 ; 93.0)† |  |
| 60–89 days | 20 | 26 | 27 | 32 | 57.0 (-83.4 ; 90.0) | 47.3 (-132 ; 88.1)† |  |
| ≥90 days | 7 | 8 | 32 | 36 | NA | NA |  |

IE: Immunization Effectiveness; CI: confidence intervals; NA: not applicable.

* The number of total cases and controls in time since immunization intervals include the total number of non-immunized infants plus the number in each time since immunization interval, respectively, for cases and controls.

**Logistic regression, adjusted by sex, risk factors, week of admission (NCS with 2 inner knots), age at admission (NCS with 2 inner knots) and RSV hospitalization rate (per 100,000 population, by region and epidemiological week) (NCS with 2 inner knots).

†Estimate based on events-per-variable ratio between 5 and 10.

1. Exclusion of infants who received nirsevimab within five days to symptom onset (N = 552 children)

|  | **Cases** | | **Controls** | | **Crude IE (95% CI)** | **Adjusted IE****  **(95% CI)** |  |
| --- | --- | --- | --- | --- | --- | --- | --- |
|  | **Immunized** | **Total** | **Immunized** | **Total** |  |  |  |
| **Overall** | 137 | 199 | 304 | 353 | 64.2 (43.8 ; 77.1) | 64.8 (44.2 ; 77.9) |  |
| **Time since immunization** |  |  |  |  |  |  |  |
| 0-34 days | 27 | 90* | 91 | 141* | 78.8 (61.0 ; 88.5) | 78.9 (55.2 ; 90.0) |  |
| 35-69 days | 41 | 104* | 78 | 128* | 67.1 (42.0 ; 81.3) | 69.2 (43.2 ; 83.4) |  |
| ≥70 days | 69 | 132* | 135 | 185* | 49.8 (16.2 ; 69.9) | 49.2 (9.4 ; 71.5) |  |
| **Age at admission** |  |  |  |  |  |  |  |
| <60 days | 38 | 60 | 116 | 132 | 78.2 (52.2 ; 90.1) | 79.0 (53.5 ; 90.5) |  |
| 60–119 days | 43 | 57 | 76 | 85 | 67.4 (13.8 ; 87.7) | 61.0 (-4.0 ; 85.4) |  |
| 120–179 days | 31 | 43 | 46 | 52 | 60.5 (-20.9 ; 87.1) | 57.2 (-32.9 ; 86.2) |  |
| ≥180 days | 25 | 39 | 66 | 84 | 37.1 (-53.5 ; 74.2) | 42.1 (-46.2 ; 77.1) |  |
| **Catch-up immunization** | | | | | | | |
| **Overall** | 69 | 102 | 124 | 147 | 58.8 (21.3 ; 78.5) | 63.6 (26.9 ; 81.9)† |  |
| **Time since immunization** |  |  |  |  |  |  |  |
| 0-34 days | 3 | 37* | 14 | 37* | NA | NA |  |
| 35-69 days | 12 | 46* | 21 | 44* | 74.0 (30.9 ; 90.2) | 80.0 (42.2 ; 93.1)† |  |
| ≥70 days | 54 | 88* | 89 | 112* | 47.0 (-6.7 ; 73.7) | 53.6 (1.7 ; 78.1)† |  |
| **Age at admission** |  |  |  |  |  |  |  |
| <60 days | 0 | 0 | 2 | 2 | NA | NA |  |
| 60–119 days | 16 | 23 | 23 | 25 | 83.3 (3.8 ; 97.1) | 81.1 (-27.7 ; 97.2)† |  |
| 120–179 days | 28 | 40 | 33 | 36 | 77.2 (8.3 ; 94.3) | 74.8 (-4.8 ; 93.9)† |  |
| ≥180 days | 25 | 39 | 66 | 84 | 38.2 (-51.0 ; 74.7) | 43.9 (-43.2 ; 78.0)† |  |
| **At-birth immunization** | | | | | | | |
| **Overall** | 68 | 97 | 180 | 206 | 67.4 (38.7 ; 82.7) | 69.5 (41.6 ; 84.0)† |  |
| **Time since immunization** |  |  |  |  |  |  |  |
| 0-34 days | 24 | 53* | 77 | 104* | 76.1 (49.0 ; 88.8) | 81.3 (55.1 ; 92.2)† |  |
| 35-69 days | 29 | 58* | 57 | 84* | 62.3 (21.4 ; 81.9) | 64.6 (20.4 ; 84.3)† |  |
| ≥70 days | 15 | 44* | 46 | 73* | 58.2 (3.0; 82.0) | 35.2 (-106 ; 79.6)† |  |
| **Age at admission** |  |  |  |  |  |  |  |
| 0–29 days | 16 | 27 | 47 | 54 | 79.7 (34.2 ; 93.7) | 88.0 (54.5 ; 96.8)† |  |
| 30–59 days | 22 | 33 | 67 | 76 | 76.1 (31.4 ; 91.7) | 80.3 (40.9 ; 93.4)† |  |
| 60–89 days | 21 | 27 | 32 | 37 | 60.9 (-61.0 ; 90.5) | 51.9 (-106 ; 88.7)† |  |
| ≥90 days | 9 | 10 | 34 | 39 | NA | NA |  |

IE: Immunization Effectiveness; CI: confidence intervals; NA: not applicable.

* The number of total cases and controls in time since immunization intervals include the total number of non-immunized infants plus the number in each time since immunization interval, respectively, for cases and controls.

**Logistic regression, adjusted by sex, risk factors, week of admission (NCS with 2 inner knots knots), age at admission (NCS with 2 inner knots) and RSV hospitalization rate (per 100,000 population, by region and epidemiological week) (NCS with 2 inner knots).

†Estimate based on events-per-variable ratio between 5 and 10.
